# Increasing Dengue Burden and Severe Dengue Risk in Bangladesh: An Overview

**DOI:** 10.3390/tropicalmed8010032

**Published:** 2023-01-03

**Authors:** Mohammad Enamul Hoque Kayesh, Ibrahim Khalil, Michinori Kohara, Kyoko Tsukiyama-Kohara

**Affiliations:** 1 Department of Microbiology and Public Health, Faculty of Animal Science and Veterinary Medicine, Patuakhali Science and Technology University, Barishal 8210, Bangladesh; 2 Department of Livestock Services, Ministry of Fisheries & Livestock, Government of the Peoples Republic of Bangladesh, Dhaka 1215, Bangladesh; 3 Department of Microbiology and Cell Biology, Tokyo Metropolitan Institute of Medical Science, Tokyo 156-8506, Japan; 4 Transboundary Animal Diseases Centre, Joint Faculty of Veterinary Medicine, Kagoshima University, Kagoshima 890-0065, Japan

**Keywords:** dengue virus, dengue, epidemiology, vector control, challenges, remedies

## Abstract

Dengue is a prevalent and rapidly spreading mosquito-borne viral disease affecting humans. The geographic range of dengue is expanding, and much like in many other tropical regions of the world, dengue has become a major public health issue in Bangladesh. Until a large epidemic dengue outbreak in 2000, sporadic outbreaks have occurred in Bangladesh since 1964. After 2000, varying intensities of dengue activity were observed each year until 2018. However, in 2019, Bangladesh experienced the largest dengue epidemic in its history, with 101,354 dengue cases and 164 dengue-related deaths. Notably, this outbreak occurred in many regions that were previously considered free of the disease. As of 10 December 2022, a total of 60,078 dengue cases and 266 dengue-related deaths were reported in Bangladesh, with the 2022 outbreak being the second largest since 2000. There is an increased genetic diversity of the dengue virus (DENV) in Bangladesh and all four DENV serotypes are prevalent and co-circulating, which increases the risk for severe dengue owing to the antibody-dependent enhancement effect. Vector control remains the mainstay of dengue outbreak prevention; however, the vector control programs adopted in Bangladesh seem inadequate, requiring improved vector control strategies. In this review, we provide an overview of the epidemiology of DENV infection and the risks for a severe dengue outbreak in Bangladesh. Additionally, we discuss different dengue vector control strategies, from which the most suitable and effective measures can be applied in the context of Bangladesh for tackling future dengue epidemics.

## 1. Introduction

**Figure 1:**
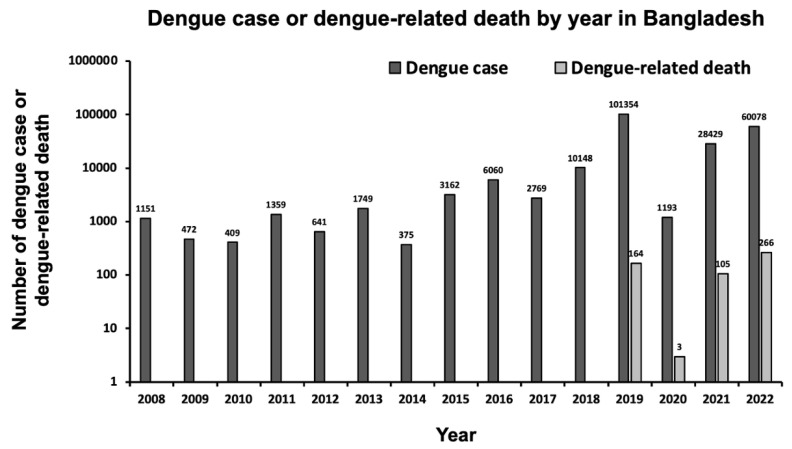
Number of reported dengue case and dengue-related death per year in Bangladesh between 2008 and 10 December 2022.

Dengue is a public health problem in many tropical and subtropical countries, particularly in urban and semi-urban areas, where most outbreaks have been reported [1]. Many factors have influenced the global rise of dengue, including population growth, high population density, unplanned rapid urbanization and construction, climate change, absence of reliable piped water, and ineffective vector control strategies [2,3,4,5]. The rapid global spread of dengue is also associated with increased human mobility through air travel [6,7]; 75% of the global dengue burden lies in Southeast Asia and the Western Pacific region [8]. The incidence of overall global dengue virus (DENV) infection has also increased rapidly in the last two decades; 505,430 cases were reported in 2000, while over 2,400,138 and 3,312,040 cases have been reported in 2010 and 2015, respectively. The number of deaths has also increased from 960 to more than 4032 between 2000 and 2015 [1]. Each year, an estimated 100–400 million infections occur, and over 80% of these infections are generally mild and asymptomatic [1]. In line with global trends, the incidence of dengue has also dramatically increased in Bangladesh. A recent study estimated that in Bangladesh, 40 million [range: 34.3–47.2] people are infected nationally, with 2.4 million [range: 1.3–4.5] annual infections [9]. The first dengue outbreak in Bangladesh was reported in 1964 in East Pakistan, and the term Dacca fever was coined [10,11]. The first official dengue outbreak in Bangladesh was reported in 2000, with 5551 cases and 93 deaths reported [12]. Since then, dengue has become endemic in Bangladesh. In 2018, more than 10,000 cases of dengue were reported for the first time. Notably, in 2019, Bangladesh witnessed one of the largest dengue epidemics in its history with 101,354 dengue cases and 164 dengue-related deaths being reported (Figure 1) [13]. In 2020, Bangladesh reported 1405 dengue cases and only three confirmed dengue-related deaths (Figure 1) [14]. In 2021, 28,429 dengue cases and 105 dengue-related deaths were reported (Figure 1). In the year 2022, an increasing trend of dengue outbreaks was observed in many countries, including Bangladesh. As of 23 November 2022, a total of 3,643,763 dengue cases and 3380 dengue-related deaths were reported globally [15]. As of 10 December 2022, a total of 60,078 dengue cases and 266 dengue-related deaths were reported in Bangladesh, and the 2022 outbreak is the second-largest outbreak since 2000 (Figure 1) [16].

**Figure 2:**
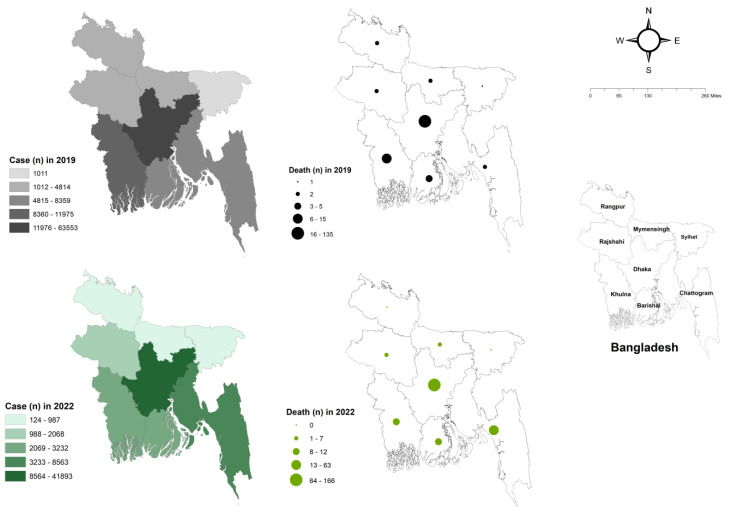
Dengue case and dengue-related death reported in 2019 and 2022 in different divisions of Bangladesh. The number of dengue case and dengue-related death in the years 2019 and 2022 (as on 10 December 2022) in different divisions (Dhaka, Chattogram, Rajshahi, Khulna, Sylhet, Barishal, Rangpur, and Mymensingh) of Bangladesh are shown. ArcGIS-ArcMap version 10.2 (ESRI, Redlands, CA, USA) was used to produce a choropleth map of the frequency of dengue cases and a graduated symbol map of the number of deaths in different divisions (Dhaka, Chattogram, Rajshahi, Khulna, Sylhet, Barishal, Rangpur, and Mymensingh) of Bangladesh.

Importantly, regional variation in dengue occurrence was observed both in 2019 and 2022 (Figure 2). In particular, the Directorate General of Health Services (DGHS), Bangladesh reports data on dengue cases and deaths separately for Dhaka City and the Dhaka Division excluding Dhaka City; however, to show division-wise dengue cases and death occurrence, we used Dhaka Division data that also included data for Dhaka City (Figure 2). In 2019, the highest occurrence of dengue cases was observed in Dhaka Division, followed by the Khulna, Chattogram, Barishal, Rajshahi, Mymensingh, Rangpur, and Sylhet divisions (Figure 2). In 2022, the highest occurrence of dengue cases was again observed in the Dhaka Division, followed by Chattogram, Khulna, Barishal, Rajshahi, Mymensingh, Rangpur, and Sylhet, suggesting that Dhaka Division—in particular, Dhaka City—was the center point for dengue outbreak. The number of dengue-related deaths increased in 2022 compared to those in 2019 (Figure 1). However, it is likely that the coronavirus disease 2019 (COVID-19) pandemic may have hampered dengue case reporting, since the first reported COVID-19 case in Bangladesh was on 8 March 2020 [17]. Notably, compared to the previous four years (2018–2021), as on 24 July 2022, there was an acute surge in dengue, resulting in 7687 confirmed dengue cases and six dengue-related deaths (case fatality rate, 0.08%) in the Rohingya refugee/Forcibly Displaced Myanmar Nationals camps in Cox’s Bazar district, Bangladesh [18].

The DENV, the causal agent of dengue, is a positive-sense, single-stranded RNA virus with a genome size of 10.7 kb and belongs to *Flavivirus*, in the family Flaviviridae [19]. *Flavivirus* contains many other important pathogenic viruses, including yellow fever virus, Japanese encephalitis virus, tick-borne encephalitis virus, Usutu virus, West Nile virus, and Zika virus [20]. The DENV genome encodes three structural proteins, namely the capsid (C), membrane (M), and envelope (E) proteins, and seven nonstructural proteins, including NS1, NS2A, NS2B, NS3, NS4A, NS4B, and NS5 [19,21]. There are four DENV serotypes, DENV-1, DENV-2, DENV-3, and DENV-4, which are genetically related but antigenically distinct [2]. All four DENV serotypes emerged from sylvatic strains in the forests of Southeast Asia [22]. Recently, DENV serotype 5 was reported to be present only in the sylvatic cycle [23].

The DENV is spread via a human-mosquito-human cycle through the bites of infectious female *Aedes* mosquitoes, mainly *Aedes aegypti*, and to a lesser extent, *Aedes albopictus* [24]. Female mosquitoes acquire the virus while feeding on the viremic blood of a DENV-infected human. Vertical transmission of DENV in mosquitoes between generations has also been reported; however, its significance remains unclear [25,26]. Both *Ae. aegypti* and *Ae. albopictus* mosquitoes feed during the daytime from morning until dusk; however, nighttime biting has also been reported in *Ae. albopictus* [27]. DENV causes a spectrum of illnesses in humans, ranging from asymptomatic to mild fever, as well as dengue hemorrhagic fever and dengue shock syndrome, which are often fatal if not properly treated [28]. The World Health Organization (WHO) revised dengue case classification, where severe dengue cases were reported to contain severe plasma leakage leading to dengue shock syndrome and fluid accumulation with respiratory distress, severe bleeding, and severe organ involvement, including the liver (AST or ALT ≥ 1000) and central nervous system, which may cause impaired consciousness, and other organs including heart, and occasional death may occur [29,30]. In SAARC (South Asian Association for Regional Cooperation) countries, the case fatality has been reported to be 1.9% of dengue cases [31].

Until the advent of a successful vaccine candidate, a continued efficient and successful vector control program is critical for dengue control and prevention. To control the spread of the DENV, vector control is essential and remains the primary tool to prevent DENV infections [32]. A human/mammalian virus, DENV can infect mosquito midgut cells and other tissues and spread to the salivary glands, and during feeding, infected mosquitoes transmit DENV to humans [33]. The DENV is transmitted between humans and *Ae. aegypti* and *Ae. albopictus* mosquitoes, which are widely distributed in Bangladesh [34]. Risk of mosquito infection is positively associated with high viremia and high fever in the patient. In contrast, DENV-specific high-level antibodies are associated with a decreased risk of mosquito infection [35]. Mosquito salivary gland extract has also been reported to exacerbate dengue pathogenesis [36]. Therefore, the implementation of vector control strategies is important for controlling DENV infection [37,38].

Although protection against homologous reinfection is lifelong, only short-term protection can be obtained against heterologous infection [39,40]. Moreover, subsequent heterologous infection may threaten severe dengue development through antibody-dependent enhancement (ADE) [41,42]. Therefore, vaccines capable of providing long-term protection against each of the four DENV serotypes are critical for preventing ADE-mediated severe dengue and for controlling DENV infections [43]. In the absence of a panserotype preventive dengue vaccine, the prevention and control of DENV infections might largely depend on effective vector control measures [1]. From this point of view, an understanding of the epidemiology of DENV infections and the prevalence of vectors and their control strategies are important for the control of dengue outbreaks. Therefore, in this study, we discuss our current understanding of the epidemiological pattern of DENV infection in Bangladesh and the risks for the development of severe dengue owing to ADE effects, which is caused by heterologous infection. Additionally, we also discuss different vector control strategies for the control and prevention of future dengue epidemics.

## 2. Epidemiology of DENV Infection and Correlation of Serotypes with Disease Severity

Dengue appears as a major cause of morbidity and mortality in Bangladesh [10,44]. According to the dengue risk level set by the Centers for Disease Control and Prevention (USA), Bangladesh is at risk at a frequent/continuous level [45]. Dengue outbreaks associated with the four different serotypes display distinct epidemiological patterns. During the first dengue epidemic in Bangladesh in 2002, DENV-3 was identified as the main serotype [46]. However, in the years 2013–2016, DENV-1 and DENV-2 were the predominant serotypes in Bangladesh [47]. In 2017, DENV-2 was the predominant serotype (91.3%) in Dhaka City [48]. A recent study phylogenetically analyzed samples collected during the 2018 outbreak in Bangladesh and found cocirculation of serotypes 2 (54%) and 3 (46%) [49]. In 2018, among 127 cases, DENV-2 was reported as the predominant serotype (40.95%), followed by DENV-3 (33.07%) and DENV-1 (25.98%) [48]. During the 2019 and 2021 epidemics, DENV-3 was the predominant serotype [48,50]. Notably, serotyping results of 10 samples obtained from the Forcibly Displaced Myanmar Nationals camps in Cox’s Bazar District, Bangladesh, were processed at the Institute of Epidemiology, Disease Control and Research (IEDCR) reference laboratory in the capital Dhaka; DENV-3 (five samples) and DENV-2 (three samples) were identified, whereas two samples showed inconclusive results [18].

Based on data from January 2008 to December 2019, the median number of patients admitted to a hospital with dengue fever per year was 1554 (range: 375–101,354) [51]. Severe dengue cases are usually observed when more than one DENV serotype is present in endemic areas of the country [21,52]. As infection with four different DENV serotypes (DENV-1, DENV-2, DENV-3, and DENV-4) has been reported in Bangladesh, which suggests co-circulation of all four DENV serotypes [49,53,54,55], the risk of reinfection with heterologous DENV may be enhanced [41], constituting a major threat for severe dengue outbreaks in the near future in Bangladesh.

However, there is mixed reporting of the association of DENV serotypes and disease severity. Serotype DENV-2 was reported to be marginally associated with more severe dengue disease in a pediatric population in Thailand [56]. Another study found DENV-1 as the serotype mostly associated with severe dengue in mono-infected patients and DENV-1/DENV-2 amongst the co-infected patients [57]. However, DENV-3 was also reported to show the greatest percentage of severe cases in primary infection in the Southeast Asian region [58]. Secondary infection with a heterotypic serotype is well-known to enhance the risk of severe dengue owing to ADE effects, as observed in both human and animal models [41,59,60]. Secondary infection with DENV-2, DENV-3, and DENV-4 was reported to increase the risk of severe dengue infections in Southeast Asian regions, and DENV-2 and DENV-3 presented an increased risk of severe dengue in non-Southeast Asian regions [58]. However, a recent study reported no association between the severity of disease and serotype found [61].

However, the increased genetic diversity of DENV in Bangladesh [49,55] portends the severe consequences of an epidemic dengue outbreak. In addition, when dengue patients recover from infection with one serotype, they can develop solid immunity against that particular serotype, but they are not able to avoid infection by other serotypes. These heterotypic infections may lead to the development of severe dengue as well as increased mortality [41,62,63,64,65]. Therefore, a continuous virological surveillance is critical for early warning of new serotype emergence in the circulation as well as for public health preparedness [66].

## 3. Vector Control

Dengue is one of the important vector-borne diseases that is prevalent globally. Both biotic factors such as feeding, predation, and intra- and inter-specific competition and abiotic factors, including water salinity, pH, conductivity, and total dissolved solids, can influence immature mosquito development [67,68]. Water with < 0.5 parts per thousand (ppt), 0.5–30 ppt, and > 30 ppt salt are termed fresh, brackish, and saline water, respectively [69]. The larvae of *Ae. aegypti* develop in fresh water, however, tolerance of this species in brackish water has also been observed [69,70]. *Ae. aegypti* and *Ae. albopictus* larvae were reported to be found in brackish water containing salinity from 2 to 15 ppt in discarded containers, abandoned fishing boats and unused wells in coastal peri-urban environment [69]. It has been observed that growth rates of *Ae. aegypti* decrease with increasing salinity [68]. However, it is expected that salinity-tolerant *Ae. aegypti* might have the ability to reduce the efficacy of insecticides, which may make it difficult to control arboviral diseases [71]. Expansion of coastal brackish water habitats and their neglect for control measures might influence the spreading of salinity-tolerant *Ae. aegypti* and genes for salinity tolerance [71]. However, the impact of salinity on *Aedes* mosquito in coastal areas of Bangladesh remains to be investigated.

Effective vector-based DENV prevention involves initiating control measures, including source reduction, destruction of larvae by larvicide treatment before the beginning of the mosquito season, and killing adult mosquitoes using adulticides. Moreover, recent studies showed that mass trapping can also be used for the control of *Ae. aegypti* populations [72] and should help in dengue control. It is well accepted that sleeping under a bed net can help in preventing contact with dengue vectors [73]. Moreover, there are national guidelines for prevention of other mosquito-borne diseases, including dengue, in Bangladesh, and their proper implementation should reduce incidences of dengue outbreak [74]. The major vector control strategies targeting *Ae. aegypti* and *Ae. albopictus* include physical, chemical, and biological control.

Physical control: Habitat management works by reducing mosquito breeding sites. Vehicle parts and discarded construction materials were reported as the most efficient producers of *Aedes* mosquitoes, and a significantly (*p* < 0.05) high presence of *Aedes* mosquitoes was reported in low socio-economic zones of Dhaka [75]. Tires, plastic buckets, plastic drums, and coconut shells were also reported as the most prevalent container types used as *Aedes* habitats in Bangladesh [34]. Therefore, proper use, disposal, and recycling of the containers are critical for reducing the risk of DENV transmission [75]. As *Aedes* larvae are born in stagnant water, every resident should take care so that water does not accumulate on the roof of the house, in the courtyard, or even in flower tubs.

Based on meta-analysis, a previous study showed that house screening and combining community-based environmental management and water container covers can significantly reduce dengue risk [76]. In another study, house screening was also reported as a feasible alternative approach for preventing human-vector contact, and may help to sustain long-term suppression of household infestations of *Ae. aegypti* [77]. The significance of community-based programs for elimination of dengue mosquitoes has been demonstrated to be effective in different areas, including Kerala, India [78], Mexico [79], and Cuba [80]. Therefore, a sustained community involvement is essential to improve vector control efforts substantially [1].

Chemical control: Chemical control is achieved using either synthetic or natural insecticides, and the latter is preferable. Insecticides belonging to the chemical classes of pyrethroids, carbamates, organophosphates, and organochlorines can be used for chemical control of mosquitoes [81]. Insecticides can be used to target both adults and larvae in the form of space treatment, indoor residual spraying, insecticide-treated bed nets, and as larvicides [82]. Fogging and spraying insecticides targeting adults and larvae are commonly practiced in Dhaka City; however, these approaches have not been demonstrated to be effective in mosquito control [83,84]. Although the epidemiological importance of thermal fogging remains unclear, peridomestic space spraying is considered as part of an integrated vector management strategy [85], and its effectiveness should be measured in terms of the impact on both adult and immature mosquito populations and disease transmission. Notably, insecticide resistance has been reported in a number of different countries [82,86] including Bangladesh [87]; therefore, the effectiveness of using particular insecticides at the recommended dosage should be investigated regularly. In particular, a recent study conducted in Sri Lanka reported that a much higher concentration of temephos is required than the WHO recommendation (0.012 mg/L) for controlling *Ae. aegypti* and *Ae. albopictus* [88]. Although the physical integrity of the long-lasting insecticidal net can be compromised over time, the remaining chemical effect can still contribute to killing/repelling mosquitoes, suggesting that fitting long-lasting insecticidal net as screens on doors/windows should have a significant impact on the indoor prevalence of adult *Ae. aegypti* [89]. Insecticide-treated screening could be a promising approach, as it targets adult mosquitoes and leads to reduced human-mosquito contact.

Biological control: Biological control may play a significant role in preventing the spread of DENV infections. *Wolbachia is* a bacterium commonly found in many species of insects, including dragonflies, butterflies, and moths, but does not occur naturally in *Ae. aegypti* [90]. Although the *Wolbachia*-mediated antiviral mechanism is not well-understood, several studies have shown that *Ae. aegypti* infected with some strains of *Wolbachia* can provide resistance to several arbovirus infections, including DENV [91,92,93,94,95,96]. *Wolbachia* has been suggested to induce a form of embryonic death due to sperm-egg incompatibility, called cytoplasmic incompatibility [97,98], when *Wolbachia*-infected males mate with uninfected females or females infected with an incompatible *Wolbachia* strain. The *Wolbachia*-mediated biological method of dengue control has been found to be successful in reducing dengue incidence in several areas of dengue-prone countries, including Australia, Malaysia, Vietnam, and Indonesia [95,99]. This biological approach to *Wolbachia*-mediated control of DENV infection remains to be investigated in Bangladesh, however it would be a promising option to control dengue in Bangladesh [95,99].

Use of larvivorous fish, such as *Poecilia reticulata* and *Gambusia affinis*, could be another approach for dengue vector control [100,101,102]. However, its efficacy as a single agent or in combination with other control measures, including use of larvicides, requires further investigation to reach a conclusion [100,103]. Biological control of dengue vectors by using copepods such as *Macrocyclops albidus, Mesocyclops* spp. have been reported in several studies [104,105,106], however, this remains to be investigated in Bangladesh.

The larvicidal toxins produced by *Bacillus thuringiensis* subspecies *israelensis* (Bti) and *Lysinibacillus sphaericus* have been used in biological control of *Aedes* larvae [106]. One recent study reported a long-lasting biological larvicide composed of Bti mixed with water-soluble polyethylene glycols and water-insoluble hexadecanol, which displayed very good efficacy in the control of the dengue vector mosquito *Ae. albopictus* [107], which could be developed as an effective measure for dengue vector control. Control of *Aedes* mosquito larvae with a carnivorous aquatic plant of North America, *Utricularia macrorhiza*, has been reported [108], which requires further investigation. Moreover, native carnivorous aquatic plant targeting *Aedes* larvae could also be investigated in Bangladesh.

Use of sterile insect technique (SIT) is a promising strategy that helps in prevention and control of mosquito-borne diseases [109]. As the name indicates, SIT disrupts the target organism’s reproductive cycle, where mass-reared male mosquitoes, sterilized using X-ray or gamma-ray ionization, are released, which helps in suppressing the fecundity rate in female mosquitoes resulting reduced vector density in urban environments and this vector-borne disease transmission [110,111,112].

However, the combination of different vector control strategies is suggested to be more effective than any single approach [113]. Notably, a comparative analysis of the relative efficacy of vector-control strategies revealed that adulticide application is the most effective method, which is followed by reducing exposure to mosquito bites, locating and destroying breeding places and, finally, larvicides use [114]. The strengths and limitations of different dengue vector control strategies are listed in Table 1.

## 4. Discussion

The abundance and transmission potential of *Ae. aegypti* are influenced by temperature and precipitation [124], suggesting *Aedes* mosquito as a climate-sensitive vector. The climate conditions in Bangladesh are becoming increasingly favorable for the transmission of vector-borne disease such as dengue [125]. A previous study analyzed 40,476 cases between 2000 and 2017, and observed that 49.73% of cases were reported during the monsoon season (May–August) and 49.22% during the post-monsoon season (September–December) [126]. However, since 2014, dengue cases have been reported during the pre-monsoon season, suggesting a change in dengue occurrence, which could be owing to the effects of climatic change in Bangladesh. Some risk factors affecting dengue outbreaks in Dhaka, including storage of water in household utilities and poor water management, which could be used as mosquito development sites were identified [127]. A recent year-round surveillance study found that the abundance of *Aedes* mosquito larvae in Dhaka varied in different months, and the highest and lowest number of *Aedes* larvae were found in the months of June and February, respectively [128]. A recent study estimated that 24% of the Bangladesh population has been infected by dengue in their lifetime; however, this varied from 3% in the north to >80% in Dhaka [9].

As Dhaka City remains the hotspot for the upsurge in dengue cases across the country, the city corporations of Dhaka such as Dhaka North City Corporation (DNCC) and Dhaka South City Corporation (DSCC) should make efforts to involve its residents in community-based programs for elimination of dengue mosquitoes to control dengue outbreaks. Moreover, in Bangladesh, dengue cases are recorded only by the passive surveillance of the disease where only hospitalized patients are officially counted and notified [129,130], which might impede the dengue control program in Bangladesh because of underestimation of the true dengue burden in Bangladesh. Many asymptomatic or mild cases of dengue are not hospitalized and remain uncounted. To obtain the actual number of dengue cases, both active and passive surveillance are important. Therefore, a sustained and strengthened surveillance system is essential for the early detection and isolation of DENV-infected patients to limit the spread of the infection.

Although the DNCC and DSCC have adopted several initiatives such as the opening of control rooms for conducting special anti-mosquito combing operations, including awareness building programs among residents, strengthening dengue surveillance programs to destroy the breeding sites of *Aedes* mosquitoes, and providing free dengue tests and advice [131,132], the vector control program needs to be further enhanced for effective vector control, as the dengue cases are still on the increase. Although previous dengue outbreaks (from 2000 to 2018) were mainly centered in Dhaka, the 2019 and current 2022 outbreaks spread across the country, including all divisional cities. Therefore, the country should remain prepared for an immediate response with an improved, rapid diagnostic and continuous monitoring system, which is likely to limit the spread and impact of the infection [133]. Moreover, an early detection of disease progression associated with severe dengue is important for proper medical care, which may reduce fatality rates of severe dengue to below 1% [1].

To control mosquitoes, insecticides are being used in Dhaka City by the city corporation authority, but this is not enough, as DENV infection rates are still increasing, which may be due to insecticide resistance in mosquito populations [87]. Moreover, the challenge of emergence and spreading of salinity-tolerant *Aedes* mosquitoes that might show potential to reduce the efficacy of insecticides should be investigated. Although it has been reported that adulticide application is the most effective dengue vector control method [114], it is crucial to identify the most effective interventions for vector control in the context of Bangladesh and how this can be adopted for the successful implementation of the program.

It is believed that dengue patients who have recovered from infection with one serotype can develop life-long immunity against that particular serotype, but upon exposure to other DENV serotypes, reinfection may occur. Co-circulation of multiple DENV serotypes enhances the risk of secondary infection with heterologous serotypes, which may increase the risk of developing severe dengue owing to the ADE effect [41,62,63,64,134]. Moreover, it has been reported that viral titers and serotypes also correlate with increased disease severity [134]. As all four serotypes co-circulate in Bangladesh [49,53,54,55,135], the risk of reinfection with heterotypic serotypes is likely to result in severe dengue. Compared to previous years, in the year 2022, the dengue-related deaths were observed to be the highest in number, and the association of ADE effects owing to heterotypic infections could not be excluded.

## 5. Conclusions

Managing dengue outbreaks in tropical countries, including Bangladesh, where temperatures remain favorable for mosquito breeding and viral replication throughout the year, is a big challenge. High population density, rapid and unplanned urbanization, inadequate housing, insufficient water, sewage, waste management, and favorable climatic conditions for the propagation of *Ae. aegypti* and *Ae. albopictus* mosquitoes are likely to increase the dengue burden in Bangladesh [136]. Therefore, controlling the mosquitoes early might be more efficient in limiting the dengue outbreaks in Bangladesh. While vector control methods are supposed to reduce the dengue burden, conclusive evidence is lacking for the effectiveness of any dengue vector control method [76], requiring further investigations to evaluate and compare methods to optimize cost-effective dengue prevention. Increasing community awareness is also important, which can be done through local visits by community healthcare workers, radio broadcasts with public/religious leaders and healthcare professionals to encourage the use of preventive methods, and TV as well as social media, particularly in urban areas. To date, adulticide application is considered as the most effective dengue vector control method; however, the efficacy of adulticides should be routinely confirmed, as resistance may develop. It is also important to identify the etiology and predominant serotype of an outbreak, describe the clinical presentation, and identify the factors associated with dengue. Although an efficient vector control strategy can limit the spread of a dengue outbreak, development of a universal dengue vaccine that is equally protective against all serotypes should be the focus of dengue prevention moving forward.

## Figures and Tables

**Table 1:** Comparison of different major vector control strategies targeting *Aedes aegypti* and *Aedes albopictus.*

Vector Control Methods	Application to Mosquitoes or Breeding Sites/Habitats	Strengths	Limitations
Physical control: Habitat management	Can be applied on a wide range of artificial containers	Proper habitat management may prevent or reduce the breeding of *Aedes* mosquitoes in the used tires, discarded containers, flowerpots, etc. [115]	Requires continuous surveillance for habitat removal
Chemical control: insecticides, larvicides	Can be used against adult mosquitoes and larvae including as space treatment, indoor residual spraying and insecticide-treated bed nets [106]. Can be used in small water-storage containers [116].	Mainstay of vector control worldwide, playing a major role in the prevention and control of vector-borne diseases, including dengue [117].	Insecticide resistance may compromise vector control efficacy [117], requiring resistance monitoring systems; might not be ecologically friendly
Biological control: *Wolbachia*-mediated biological method	Release of *Wolbachia*-infected mosquitoes to the local mosquito populations [118,119].	*Wolbachia*-mediated dengue vector control is novel, economic, and more ecologically friendly than using pesticides [99,106]. It can induce complete cytoplasmic incompatibility [92].	Transmission may not occur because some of the mosquitoes may not live for a period longer than extrinsic incubation period of arboviruses [92]. Further studies are warranted for confirmation of dengue vector control for the effectiveness of the method
Sterile insect technique (SIT)	Applied to mass-reared male mosquitoes to make the males sterile [112]	Could be used as a powerful complement to most commonly used approaches because of its ecologically benign, specific, and non-persistent nature in the environment once releases are stopped [120]	Manual separation of males and females is required [106]; successful reduction in populations only achieved in a few instances [121]
Use of larvivorous fish *Poecilia reticulata* (guppy) and *Gambusia affinis* (mosquito fish)	Can be used in water storage containers [101]	It helps in the reduction of immature larvae [100]	The chance of off-target effects such as targeting of other arthropod species cannot be ignored.
Use of copepods (mainly *Mesocyclops* and *Macrocyclops* species)	Can be applied to control of container-inhabiting mosquitoes [122]	High predation efficiency [123]	Most effective against first instar larvae [106]
Use of biological larvicides: *Bacillus thuringiensis* subspecies *israelensis* (Bti)	Can be applied to water-storage containers [116]	Little or no impact on non-target organisms and no accumulation in the environment	Difficulty in maintenance, storage, and transportation [106]

## Data Availability

Not applicable.
